# Improving Person-Centredness in Integrated Care for Older People: Experiences from Thirteen Integrated Care Sites in Europe

**DOI:** 10.5334/ijic.5427

**Published:** 2020-06-26

**Authors:** Annerieke Stoop, Manon Lette, Eliva A. Ambugo, Erica Wirrmann Gadsby, Nick Goodwin, Julie MacInnes, Mirella Minkman, Gerald Wistow, Nick Zonneveld, Giel Nijpels, Caroline A. Baan, Simone R. de Bruin, on behalf of the SUSTAIN consortium

**Affiliations:** 1 National Institute for Public Health and the Environment, Bilthoven, NL; 2 Amsterdam Public Health research institute, Amsterdam UMC - VU University Amsterdam, Amsterdam, NL; 3 Scientific Centre for Transformation in Care and Welfare (Tranzo), Tilburg University, Tilburg, NL; 4 Department of Health Management and Health Economics, Institute of Health and Society, University of Oslo, Oslo, NO; 5 Centre for Health Services Studies, University of Kent, Canterbury, UK; 6 Central Coast Research Institute for Integrated Care and Population Health, University of Newcastle, AU; 7 Vilans, National Centre of Expertise in Long Term Care, Utrecht, NL; 8 TIAS School for Business and Society, Tilburg University, Tilburg, NL; 9 Care Policy and Evaluation Centre, London School of Economics and Political Science, UK

**Keywords:** older people, integrated care, person-centredness, mixed methods, implementation science, European research

## Abstract

**Introduction:**

Although person-centredness is a key principle of integrated care, successfully embedding and improving person-centred care for older people remains a challenge. In the context of a cross-European project on integrated care for older people living at home, the objective of this paper is to provide insight at an overarching level, into activities aimed at improving person-centredness within the participating integrated care sites. The paper describes experiences with these activities from the service providers’ and service users’ perspectives.

**Methods:**

A multiple embedded case study design was conducted that included thirteen integrated care sites for older people living at home.

**Results:**

Service providers were positive about the activities that aimed to promote person-centred care and thought that most activities (e.g. comprehensive needs assessment) positively influenced person-centredness. Experiences of service users were mixed. For some activities (e.g. enablement services), discrepancies were identified between the views of service providers and those of service users.

**Discussion and conclusion:**

Evaluating activities aimed at promoting person-centredness from both the service providers’ and service users’ perspectives showed that not all efforts were successful or had the intended consequences for older people. Involvement of older people in designing improvement activities could ensure that care and support reflect their needs and preferences, and build positive experiences of care and support.

## Introduction

In Europe, integrated care sites are increasingly being put in place to provide care to older people with multiple health and social care needs who live at home [1,2]. In this context, integrated care is defined as those approaches that proactively seek to structure and coordinate health and social care for older people in their home environments, centred around older people’s needs [3,4,5,6,7]. One of the main principles of integrated care is person-centredness [8,9,10,10,11,12]. The literature includes several definitions of person-centredness but a universally agreed one is lacking [13,14,15,16,17,18]. Common elements in these definitions include: 1) empowering and encouraging people to participate actively, as equal partners, in decision-making processes about their own care, and/or to manage their own health and care; 2) establishing an accommodating, cooperative and ongoing relationship between the professional, the person receiving care and the informal carer, including respectful communication and active listening; 3) having an understanding of the specific (health) concerns of the person, and their individual needs and preferences; 4) addressing the physical, cognitive, psychological and social domains of the person’s life; and 5) providing coordinated care to achieve continuity and coherence of care and support [19].

Even though integrated care appears to be a promising approach for organising services more comprehensively around the needs, preferences and capabilities of individual older people [20], effective implementation of person-centred care is still a challenge [21,22]. Involvement of older people in decision-making regarding their own care and support processes is often limited, since they are often regarded as passive recipients of care rather than active participants. This results in services that are insufficiently consistent with older people’s values and preferences for care and support [16,23]. Additionally, studies have reported difficulties with communication and information exchange between professionals and older people. Such difficulties included the lack of attentive listening, and insufficient efforts to understand older people’s individual needs [16,24,25]. Overall, successfully embedding person-centred care remains a struggle. In addition, person-centredness is a multi-dimensional concept, perceived by different actors in different ways. Some efforts made by professionals to improve person-centredness may be experienced rather differently by those receiving the care and support [26,27,28]. Therefore, the perspectives of both service providers and service users (i.e. older people and their informal carers) need to be taken into account to obtain a comprehensive and accurate picture of person-centred ways of working.

The research presented in this study aims to promote an understanding of how person-centred care is delivered in the context of integrated care, and to do so from multiple perspectives. This study was conducted within the European project SUSTAIN (Sustainable Tailored Integrated care for older people in Europe). It aimed to improve integrated care for older people living at home across different regions in Europe [7]. In the SUSTAIN project, stakeholders from established integrated care sites and researchers collaborated to develop and implement a wide variety of activities to improve different aspects of integrated care, including person-centredness. Within the above context, the objectives of this paper are: 1) to identify the activities undertaken as part of integrated care sites within SUSTAIN that aimed to promote person-centredness in care and support for older people living at home; and 2) to understand the perspectives of multiple actors (i.e. managers, health and social care professionals, older people and their informal carers) on these different activities undertaken. For this paper, we provide insights on person-centredness in the context of integrated care, and from a SUSTAIN-wide perspective. Thus, the paper’s perspective is on the overarching patterns that were identified across all the SUSTAIN sites, rather than on findings and experiences from individual sites.

## Methods

### Design and setting

In the SUSTAIN project, thirteen integrated care sites were involved, and they were located in seven countries in Europe: Austria, Estonia, Germany, Norway, Spain, the Netherlands and the United Kingdom. These sites served different target groups and provided different types of care services, including proactive primary and social care for frail older people, care for older people being discharged from hospital, care for people with dementia, and home nursing and rehabilitative care (see Table 1). Between autumn 2015 and spring 2018, SUSTAIN’s research partners supported local steering groups at the integrated care sites, consisting of representatives from different organisations (e.g. GP practice, hospital, home care organisation, social care organisation, municipality, advocacy organisation for older people), to design and implement their improvement plans. Plans consisted of sets of activities to improve different aspects of integrated care, including person-centredness, and reflected the priorities of local stakeholders [7,21]. For each of the thirteen integrated care sites, SUSTAIN’s research partners evaluated: 1) progress in implementing the different sets of activities that were part of the improvement plans, including factors that were perceived to facilitate or impede progress, and 2) the impact of the improvement plans on aspects of integrated care.

**Table 1:** Characteristics of thirteen integrated care sites participating in the SUSTAIN project.

Country	Region	Integrated care site	Type of care services

**Austria**	Vienna	Gerontopsychiatric Centre	Dementia care
**Estonia**	Ida-Viru	Alutaguse Care Centre	Home nursing and rehabilitative care
	Tallinn	Medendi	Home nursing
**Germany**	Uckermark	KV RegioMed Zentrum Templin	Rehabilitative care
	Berlin Marzahn-Hellersdorf	Careworks Berlin	Home nursing and rehabilitative care
**Norway**	Surnadal	Surnadal Holistic Patient Care at Home	Home nursing and rehabilitative care
	Søndre Nordstrand in Oslo	Søndre Nordstrand Everyday Mastery Team	Rehabilitative care and mastery of activities of daily living
**Spain**	Osona	Severe Chronic Patients/Advanced chronic disease/Geriatrics Osona	Proactive primary and intermediate care
	Sabadell	Social and health care integration Sabadell	Proactive primary care
**The Netherlands**	West-Friesland	Geriatric Care Model	Proactive primary care
	Arnhem	Good in one Go	Transitional care
**United Kingdom**	Kent	Over 75 Service	Proactive primary care
	Kent	Swale Home First	Transitional care

In SUSTAIN, a multiple embedded case study design was adopted to evaluate and compare the implementation of activities to improve integrated care across different existing integrated care sites for older people [7,29,30]. Each site served as one case study, using data triangulation to enhance the robustness of the study [29,31]. The multiple case study design enabled analysis of data across different situations (i.e. integrated care sites) to learn about improving integrated care across Europe, and enhanced understanding of the similarities and differences between the cases [30].

### Building the individual case studies

A two-step data analysis approach was adopted: 1) thirteen individual case studies were conducted and written up [32,33,34,35,36,37,38], after which 2) an overarching analysis of the case studies was conducted. The design of the SUSTAIN project is described in further detail elsewhere [7]. This paper reports on the second step, which means we focus on outcomes at a SUSTAIN-wide, overarching level.

Individual case studies were built on qualitative and quantitative data gathered from the sites using a set of qualitative and quantitative data sources. Data were collected from health and social care professionals and managers from the integrated care sites, older people receiving services from the initiatives and (informal) caregivers of these older people. More details on the set of qualitative and quantitative data sources can be found in Table 2.

**Table 2:** Qualitative and quantitative measures to monitor and evaluate improvement progress and outcomes, adapted from de Bruin et al. [7].

Data collection tool	Short description	Collection moment

**SURVEYS**		
Socio-demographics of older people (users)	Survey developed by SUSTAIN researchers including information on age, gender, education, marital status, living situation and medical conditions	Recruitment and collection took place throughout implementation period
Socio-demographics of informal carers	Survey developed by SUSTAIN researchers including information on age, gender, education, marital status, relationship and distance to user, paid work and caregiving activities	Recruitment and collection took place throughout implementation period
Socio-demographics of professionals	Survey developed by SUSTAIN researchers including information on age, gender, nationality and occupation	Collection took place at the beginning and end of implementation period
Socio-demographics of managers	Survey developed by SUSTAIN researchers including information on age, gender, nationality and occupation	Collection took place at the beginning and end of implementation period
The Person Centred Coordinated Care Experience Questionnaire (P3CEQ) [40]	Survey measuring older people’s experience and understanding of the care and support they have received from health and social care services	Recruitment and collection took place throughout implementation period
Perceived Control in Health Care (PCHC) [41]	Survey addressing older people’s perceived own abilities to organise professional care and to take care of themselves in their own homes, and perceived support from the social network	Recruitment and collection took place throughout implementation period
Team Climate Inventory – short version (TCI-14) [42,43]	Survey measuring vision, participative safety, task orientation and experienced support for innovation of the improvement team	Collection took place at the beginning and end of implementation period
**INTERVIEWS**		
Semi-structured interviews with older people and/or their informal caregivers	Interview schedule developed by SUSTAIN researchers with items regarding users’ and carers’ perceptions of and experiences with the integrated care services and the extent to which they work in a person-centred, prevention-oriented, safe and efficient manner	Recruitment and collection took place throughout implementation period
Group interview with participating health and social care professionals	Interview schedule developed by SUSTAIN researchers with items regarding professionals’ perception of and experiences with the improvement process, its facilitating and impeding factors and the extent to which it impacted person-centeredness, prevention-orientation, safety and efficiency of their way of working	Collection took place at the end of implementation period
Semi-structured interviews with managers	Interview schedule developed by SUSTAIN researchers with items regarding managers’ perception of and experiences with the improvement process, its facilitating and impeding factors and the extent to which it impacted person-centeredness, prevention-orientation, safety and efficiency of their way of working	Collection took place at the end of implementation period
**OTHER TOOLS**		
Analysis of older people’s care plans (when sites did not work with care plans, information was retrieved from clinical notes or other documentation)	Template developed by SUSTAIN researchers for predetermined content analysis of care plans, extracting information regarding needs assessments, goal-setting, medication reviews, falls, hospital and emergency admissions and advice on medication, safety and self-management	Recruitment and collection took place throughout implementation period
Sheet for efficiency indicators	Template developed by SUSTAIN researchers to collect information from staff regarding the number of hours dedicated to the improvement activities and costs of additional equipment and technology	Collection halfway through and at the end of implementation period
**PROCESS INFORMATION**		
Steering group minutes	Minutes cover processes, discussions, decisions and contextual issues impacting on outcomes and implementation progress	Collection took place throughout development and implementation periods
Field notes	Field notes cover the researchers’ notes and reflections on implementation progress	Collection took place throughout development and implementation periods

To build the individual case studies, the gathered data were analysed against predefined propositions (research questions) [29,39] using a three-staged approach, as argued by De Bruin et al. [7]. The different steps undertaken for data-analysis can be found in Table 3.

**Table 3:** Description of three-staged approach for data-analysis of the case studies.


**Step 1**	Data were analysed seperately for each individual data source (for each individual case study). For each data source, uniform templates for analysis have been generated, as appropriate for that specific data source. Qualitative data have been analysed thematically, quantitative data have been analysed using statistical methods. Appendix 1 provides the templates that have developed to analyse each data source.
**Step 2**	After analysing each individual data source, results for that source were reduced to a series of thematic statements (in case of qualitative data) and summaries (in case of quantitative data). These summaries and thematic statements were provided in English.
**Step 3**	For each case study, English thematic statements and summaries were amalgamated and underwent a process of pattern-matching across the data to gain insight into the experiences with the improvement process of the integrated care site. In order to guide this process, an analysis framework was developed (Appendix 2). Research partners analysed data against two propositions and five analytical questions: Proposition 1: Integrated care activities will maintain or enhance person-centeredness, prevention-orientation, safety, efficiency and coordination in care delivery. Proposition 2: Explanations for succeeding in improving existing integrated care sites will be identified. Analytical question 1: What seems to work and with what outcomes when making improvements to integrated care? Analytical question 2: What are the explanations for succeeding and improving integrated care sites? Analytical question 3: What are the explanations for NOT succeeding and improving integrated care sites? Analytical question 4: Are there any factors that are particularly strong in your analysis that could be seen as having an impact on integrated care improvements? Analytical question 5: What factors can you identify in your site analysis that could apply to integrated care improvements across the EU, and be transferable?


Standardised data collection tools and data analysis templates were employed by all research partners in order to ensure uniform research methods and methodological consistency across all case studies. Data collection tools and data analyses templates that were used across the case studies were developed through discussions between all involved research partners. Tools and templates were provided in English and subsequently translated into the national languages. Data collection and the initial phase (step 1) of data analysis were conducted in the national languages; the second and third steps of data analysis were conducted in English (see Table 3). Regular meetings and teleconferences took place between research partners in order to standardise methods of data collection and analyses across the different case studies.

The individual case studies were described in seven country-specific reports (written in English) by SUSTAIN’s research partners [32,33,34,35,36,37,38]. Each report was dedicated to one or two case studies from each country that participated in the SUSTAIN project and paid specific attention for the local and country context where improvement processes in the sites took place. Reports described the improvement plans of each site and the experiences with and outcomes of the improvement activities from perspectives of multiple actors (i.e. managers, health and social care professionals, older people and their informal carers).

### Overarching analysis of individual case studies

In order to address the objectives of this paper, an overarching analysis was conducted in which findings from the thirteen case studies were reviewed for evidence about how individual projects had sought to improve person-centred care. Thus, the overarching analysis aimed to identify recurring patterns and themes related to person-centred activities and experiences across all case studies, rather than on specific findings from individual sites.

The starting-point of this overarching analysis was a content analysis of the country-specific reports in which the individual case studies were described. One researcher (AS) extracted data from the reports that provided information on activities that aimed to promote person-centredness, and on experiences of managers, professionals, older people and their informal carers with these specific activities. The common elements of person-centredness (i.e. sharing power and responsibility; therapeutic relationship or alliance; patient-as-person; biopsychosocial approach; and coordinated care) were used to retrieve relevant information about promoting person-centredness from the reports [19].

The overarching content analysis was guided by the Framework Method, which supports thematic (qualitative content) analysis of textual data [44]. To conduct the analysis, a coding scheme was developed based on the objectives of this paper (i.e. deductive approach) and on the themes that emerged from reviewing the data (i.e. inductive approach) (Table 4). Two researchers (AS and ML) independently coded the data, cross-checked each other’s codings and discussed differences in order to reach consensus. Coded data were examined and the resulting findings were compared and integrated to identify recurring patterns and themes in participants’ experiences with activities that aimed to improve person-centredness. Specifically, managers’ and professionals’ views and those of older people and informal carers were compared to see whether they were in agreement with or contradicted each other. When gaps in knowledge or uncertainties based on the country-specific reports were identified, the analysis templates from individual case studies were consulted. Relevant additional data gathered from this latter step were included in the overarching analysis. Draft findings were discussed among all authors throughout the analysis process.

**Table 4:** Analysis framework used for overarching content analysis of country-specific reports.

Codes	Sub-codes

Design of health and social care delivery process	Activities
	Experiences from older people and their informal caregivers
	Experiences from health and social care professionals and managers
Staff training	Activities
	Experiences from older people and their informal caregivers
	Experiences from health and social care professionals and managers
Communication and information exchange between professionals, older people and informal carers	Activities
	Experiences from older people and their informal caregivers
	Experiences from health and social care professionals and managers
Facilitating the involvement of older people and informal carers in care and support	Activities
	Experiences from older people and their informal caregivers
	Experiences from health and social care professionals and managers

### Ethical considerations

Ethical review committees of Estonia, Norway, Spain (Catalonia) and the United Kingdom provided ethical approval of the SUSTAIN project. In Austria, Germany and the Netherlands, national standards and regulations allowed for the exemption of research activities from the need for ethics committee review. Informed consent was obtained for all study participants in all countries (including Austria, Germany and the Netherlands) prior to data collection.

## Results

The first section describes the characteristics of the participants that were involved across all individual case studies. The second section outlines the activities undertaken as part of integrated care sites within SUSTAIN that aimed at promoting person-centredness in care and support, as identified in the document analysis. Then, the third section sheds light on patterns in perspectives and experiences of multiple actors (i.e. managers, professionals, older people and their informal carers) concerning these activities. In line with the objectives of this paper, the results from the analysis (i.e. activities undertaken and recurring patterns and themes across the case studies) are reported at a SUSTAIN-wide level rather than that of individual case studies.

### Characteristics of study participants

In total, 244 older people participated across all thirteen case studies (Table 5). The proportion of females was 67%. On average, 23% of the older people were aged between 65 and 74 years, 42% were aged between 75 and 84 years, and 35% were 85 years or older. They had 5.2 medical conditions on average (range: 0–12). The proportion of older people living alone was 51%. In total, 80 informal carers were involved across all case studies. On average, 15% of the informal carers were aged between 18 and 44 years, 39% were aged between 45 and 64 years, and 46% were 65 years or older. The average proportion of female informal carers was 69%.

**Table 5:** Number of participants involved in each case study.

Integrated care site	Number of participating older people	Number of participating informal carers	Number of participating managers	Number of participating professionals

**Total**	244	80	35	205
Gerontopsychiatric Centre	7	3	2	6
Alutaguse Care Centre	28	6	1	10
Medendi	24	8	1	13
KV RegioMed Zentrum Templin	31	6	1	7
Careworks Berlin	30	7	1	14
Surnadal Holistic Patient Care at Home	29	6	2	18
Søndre Nordstrand Everyday Mastery Team	11	2	2	12
Severe Chronic Patients/Advanced chronic disease/Geriatrics Osona	19	12	3	59
Social and health care integration Sabadell	22	7	2	11
Geriatric Care Model	13	7	4	8
Good in one Go	5	6	2	8
Over 75 Service	15	5	8	31
Swale Home First	10	5	6	8

A total of 35 managers and 205 professionals participated across all case studies (Table 5). The average proportions of managers aged between 35 and 54 years or 55 years or older were 60% and 29% respectively. The large majority were female (80%). Similar to the managers, the professionals were also mostly aged between 35 and 54 years (59%) and the large majority were female (87%).

### Activities that aimed to promote person-centredness

Most sites had already implemented activities to facilitate a person-centred way of working. To further promote and improve person-centredness, they either implemented additional activities or revised existing ones.

Activities pertaining to person-centredness were found to fall into four clusters (Table 6):

1. Activities related to the design of health and social care delivery process;
2. Activities related to the organisation of training for staff;
3. Activities supporting communication and information exchange between professionals, older people and informal carers;
4. Activities facilitating the involvement of older people and informal carers in decision-making regarding their own care and support.

**Table 6:** Activities that aimed to promote person-centredness as part of integrated care sites within SUSTAIN.

Clusters of activities				

**Design of health and social care delivery process**	Working in multidisciplinary care teams	Implementing electronic care plans	Conducting comprehensive assessment of care needs	Changing the location of health and social care delivery from institutions and doctors’ offices to people’s homes
**Staff training**	Providing training on shared decision-making and person-centredness of care	Providing training on health conditions and diseases (i.e. early detection of dementia) of older people	Providing training on inter-professional communication and collaboration	
**Communication and information exchange between professionals, older people and informal carers**	Providing various options for older people and informal carers to communicate with professionals	Sharing information about available community services	Providing older people with a single point of contact as pertains to their health and social care needs	Giving older people and informal carers access to care plans
**Facilitating the involvement of older people and informal carers in care and support**	Discussing older people’s needs, preferences, goals and priorities	Involving informal carers in the care process	Empowering older people (i.e. providing them with training on shared decision-making and self-management of health)	Stimulating enablement and self-care

Some of these activities were implemented by several integrated care sites in SUSTAIN, whereas others were implemented by only one or two sites.

#### Design of health and social care delivery process

The first cluster included activities related to the design of health and social care delivery processes. Most sites implemented activities to facilitate or strengthen multidisciplinary working between professionals from health and social care organisations and/or community partners (e.g. a day centre for older people), through case conferencing meetings or multidisciplinary meetings. These meetings were organised to reflect perspectives of different professionals to support a comprehensive approach to care. Also, information about individuals’ health and wellbeing were shared in these meetings. In only a few sites, professionals had access to electronic care plans, in addition to regular staff meetings, in order to support the sharing of information about older people’s care needs among different professionals.

In almost all sites, different professionals came together to conduct a comprehensive and joint (single) assessment of older people’s care needs and to define actions to be included in care plans. The equal consideration of both the health and social perspective, and thus the recognition that they were equally valid in assessments, was expected to contribute to a thorough understanding of the broad range of older people’s needs. Only a few sites paid explicit attention to the needs for care and support of informal carers.

In several sites, the location of health and social care delivery was changed from a hospital, rehabilitation institution or doctors’ office to older people’s own homes. Services such as needs assessments and discussions about care plans, but also enablement or rehabilitation services were provided in older people’s home settings. They were thought of as comfortable and secure environments for receiving services, and (more) appropriate for conducting needs assessments. As a manager stated:

*“…those who earlier needed an institutional stay can now receive help at home. That means a lot to the user…Also, the changes reduce the number of transfers for the user [who] no longer has to first be transferred from hospital to an institution, and then home. Now, the user can go straight home.”* (Manager in Surnadal site)

#### Organisation of staff training

The second cluster of activities pertained to the organisation of training. Sites organised staff training to facilitate a more person-centred way of working. A few of them offered training to professionals on person-centred care and shared decision-making. The intent was to increase communication skills and promote active listening among professionals in order to improve shared decision-making processes. One site organised training for hospital staff on early detection of dementia. This was done to raise awareness of dementia so as to enable early recognition of symptoms and the provision of timely follow-up care and support. In another site, training on inter-professional communication and collaboration for professionals from different health and social care organisations were organised to encourage teamwork. Professionals learned to collaborate as a team and develop a network around the older person to ensure that older people are at the centre of care and support. As one involved professional said:

*“[…] collaboration with the others involved in my working area. Or OUR working area, I should say. Just that you know where to find each other. Eventually, that will benefit the patient, when you are able to make good arrangements with each other. Co-ordinate the care together, and see what is necessary from whom and tailor that to the patient’s needs.”* (Professional in Geriatric Care Model site)

#### Communication and information exchange between professionals, older people and informal carers

The third cluster included activities supporting communication and information exchange between professionals, older people and their informal carers. Most sites offered various channels (e.g. home visits, phone calls or e-mail contact) through which professionals could communicate with older people and informal carers in a quick and easy way. Professionals shared information with older people and their informal carers about services available to help them navigate easily through health and social care. For this, one of the sites focused on increasing knowledge about community services among staff. The staff were then able to inform older people about the range of services available that may support (some of) their needs. Also a single point of contact (e.g. key contact, case manager or practice nurse) for older people and their informal carers was implemented to improve information flow about available services:

*“She’s [Practice Nurse] given me a telephone number so I can get in touch if I want any help.”* (Older person in Over 75 Service site)

In one site, such information was made more accessible through the installation of a central information point (i.e. service centre).

In addition, care plans with information about older people’s functioning, care needs and goals were developed. Only in a small number of the sites were the care plans shared with older people and their informal carers. Furthermore, few provided older people and their informal carers with active roles (as delineated in the care plans) within their own capabilities, and according to their own preferences.

#### Facilitating the involvement of older people and informal carers in care and support

The fourth cluster included activities facilitating the involvement of older people and informal carers in decision-making regarding their own care and support. Professionals involved both older people and their informal carers actively in needs assessments and care planning processes, and incorporated their preferences and goals into the subsequent plans for care and support. One manager explained:

*“[…] We have been working on [incorporating] a …focus on mastery in our check-lists and facilitating [this] so that [the user] can be as independent as possible. We know that we come from a “help culture” where we rather ask ‘What do you need help with’ [since we know best] rather than what we wish to turn the question towards ‘What is important to you now?’ and hear what the user says. […]”* (Manager in Surnadal site)

In only one site, older people and their informal carers were invited to a multidisciplinary meeting to express their needs and wishes, and to validate their tailored and individualised care plan. One of the sites offered workshops to older people to empower them in shared decision-making, self-management, and identifying their needs and wishes. The workshops included content related to growing older and supported older people to reflect on their needs and preferences, together with their peers. Also one of the sites offered enablement at home (i.e. in-house reablement and rehabilitation) services to older people returning from hospital, the intention being to support them to recuperate, regain and maintain their independence at home.

### Experiences with the activities that aimed to promote person-centredness

#### Experiences with the health and social care delivery process

Overall, case studies suggested that, from the perspective of older people and their informal carers, professionals worked well together and shared information with each other about older people’s care process. In a few sites, professionals had access to electronic care plans and held regular staff meetings, all of which supported the exchange of information between professionals such that older people did not have to repeat themselves. Even so, case studies suggested that some older people felt overwhelmed or experienced mistrust from the involvement of different professionals in the care processes and the unclear delineation of their roles. There were also practical difficulties related to involvement of different professionals, as illustrated in one case study where organising multidisciplinary meetings attended by at least one health and one social care professionals proved to be challenging.

Health and social care professionals felt that conducting joint needs assessments improved person-centred working since they better understood the broad range of older people’s and their informal carers’ needs. Case studies showed that the experiences of older people and their informal carers were, however, mixed. On the one hand, many older people and informal carers were aware that a needs assessment had been carried out. They were satisfied and felt that all their needs were assessed and adequately met. Additionally, they indicated that professionals considered all domains of their lives rather than focusing exclusively on their illness or disabilities. Furthermore, in some sites, informal carers also indicated that their needs were assessed comprehensively, and that the support they received was practical and also focused on their wellbeing. On the other hand, case studies also highlighted that not all older people and informal carers had these positive experiences. Older people and informal carers sometimes indicated that their care needs and preferences were not fully assessed or assessed at an inappropriate time (for example within a few hours after hospital discharge), or that professionals focused mainly on clinical information instead of having a comprehensive approach towards their health and social care needs.

Across the different case studies, professionals indicated that providing care at home was comforting for older people, thereby contributing to a more person-centred way of working. Home visits helped professionals to better understand the older people’s (home) situation and their needs and preferences. As a result, professionals mentioned that they were able to provide advice and support that were more contextually relevant and personalised:

*“The added value of doing that [assessment] at their terrain [home] which is very important. […] really the fact that you go to his context, and you go there, and that you are really there, and that they can explain to you: “Look, this is the kitchen, this is the bathroom, I do not have that, this is what happens to me, look, there are stairs for getting into the house…”, I believe that this is an important added value, we would say, and they … I think they have perceived it in a satisfactory, very much, as a plus.”* (Professional in Sabadell site)

Older people and their informal carers mostly appreciated receiving care and support in their own home environments. However, some older people who were discharged from hospital experienced difficulties with receiving services in their home environments. They felt discharged from the hospital before they were fully prepared, which made them feel that the decisions being made were not in their best interest, as one older person said:

*“Yeah, I wasn’t 100% sure I was ready to come out.” “And was that a concern about anything in particular?” “It was just the way I was feeling in myself, I just…” “Sure, a general lack of confidence, you felt that you needed to be looked after (for) a little bit longer?” “Yeah, I just thought it was such a short (time). To me, it seems (like) quite a serious operation, and it seemed like I was just being pushed out, basically.”* (Older person in Swale Home First site)

#### Experiences with staff training

Findings from the case studies revealed that professionals had different views as to whether the training they had received improved person-centred working. Professionals indicated that training in order to increase knowledge about dementia or to promote inter-professional collaboration increased their awareness of diseases and conditions older people may suffer from, and the services available for older people. This helped them to arrange care and support services that were more aligned with older people’s functioning and needs. As one professional stated:

*“The lectures were the centrepiece.” (…) “We are much more attentive now than before. Not only the nurses are more sensitive with respect to these early signs but our physicians too. How should I put it…Yes, now, we don’t pass by if something seems strange, we look twice.”* (Professional in Gerontopsychiatric Centre site)

On the other hand, not all professionals had a positive experience and they mentioned that the training did not meet their needs. They wanted more in-depth training, focused on specific communication skills, which would be helpful in discussions with older people about their wishes and preferences and, thereby, enhance older people’s involvement in decisions about their care and support.

#### Experiences with communication and information exchange

Generally, case studies suggested that communication and relationships between staff, older people and their informal carers were experienced as positive. Overall, older people indicated that staff listened to them and treated them with kindness and respect. As quoted:

*“Are you happy with the way they treat you; the patience, with respect, are they kind…?” “Yes, yes, yes! It goes without saying.”* (Older person in Sabadell site)

They were also satisfied with the amount of time that professionals spent with them. Nonetheless, some older people felt that professionals gave them little time and attention, particularly when there were staff shortages.

Furthermore, case studies highlighted that older people and their informal carers greatly appreciated the different ways in which they could communicate with professionals (e.g. home visits, phone calls or e-mail contact). Older people and their informal carers mentioned that this improved their personal relationship with professionals. In addition, continuity of care, for instance through a single point of contact, enhanced relationships and trust between staff and older people. The single point of contact further contributed to person-centredness as older people knew who they could contact (e.g. in case of changing care needs) and felt that they were really being cared for and that their needs were being addressed very well.

*“I’ve recently had the flu unfortunately and all I had to do was to phone the surgery and I was speaking to the doctor straightaway. I had the flu jab. So she [Practice Nurse] said, ‘you know, anything you need please phone’. I was looking after [User], and [Practice Nurse] was trying to look after me.”* (Informal caregiver in Over 75 Service site)

Being transparent and clear, and using language that is easy to understand, were considered important in information sharing between professionals, older people and their informal carers. Across sites, most older people and their informal carers were satisfied with the way information was communicated to them about the available care and support services, whereas a minority indicated that information was not clearly or comprehensively shared with them.

Older people differed in their awareness of the existence of care plans, in whether or not they had access to (a printed copy of) their care plan, and in how important it was for them to have access to their care plan. Although older people and informal carers might be less concerned about this, case studies noted that lack of access to care plans meant that important information about care was not readily available to older people and their informal carers, which undermined person-centred care.

#### Experiences with the involvement of older people and informal carers in care and support

Case studies showed that professionals were positive about discussing options for care and support, and setting goals together with older people and informal carers. Professionals indicated that this helped to improve person-centred practice because they felt better able to align care and support with individual wishes and preferences. Furthermore, professionals perceived that older people highly valued being involved in planning their care and support. Case studies highlighted that the experiences of older people and informal carers were, however, more mixed than those of professionals. Many older people indicated that they discussed their needs and preferences with professionals, and they felt involved in decisions about their care. As one older person stated:

*“[…] I decide what I want [to receive] help with. They could have helped me wash, but I do not want that. I prefer that [my wife] does it.”* (Older person in Surnadal site)

However, some older people felt that decisions were made without them and they did not feel meaningfully engaged in discussions about care options, their goals and what was important to them. They found it difficult to express their needs and wishes to professionals, or to voice their concerns if they wanted changes to their care or support or felt dissatisfied.

Across different sites, also managers and professionals observed that a group of older people did not necessarily feel competent or capable of contributing to shared decision-making. This was explained by older people’s (mild) cognitive impairments or the country’s socio-historical context, where traditionally people were not used to expressing their preferences or participating in decisions (about care).

Across sites, most older people and their informal carers were satisfied with the way professionals involved informal carers in planning older people’s care and support because informal carers were often well-placed to represent older people’s needs and preferences. Still, informal carers were sometimes involved less than they had hoped for by professionals:

*“[…] But I was not asked: Do you agree that he is going to that hospital? Otherwise I would have said: Leave him here… I always asked that: I hope he can stay until there is room in that rehabilitation centre?”* (Informal caregiver in Good in one Go site)

In addition, a few older people were dissatisfied that their preferences and choices for care and support were only discussed with informal carers and not also with them, or they did not want the informal caregivers to be involved at all.

Overall, workshops that were organised for older people to empower them in shared decision-making and self-management were appreciated by them and helped them to feel supported. It also enabled them to express their wishes and preferences better, thus enhancing shared-decision making and self-management. Enablement at home for older people returning from hospital can be empowering for older people, according to the professionals involved. However, older people indicated that receiving these services can be experienced as challenging. This is particularly the case immediately after being discharged from hospital and one does not have support from close informal carers — and may be feeling anxious, exhausted and in need of greater care input.

## Discussion

### Summary of results

The aim of this paper was to identify the activities undertaken as part of different integrated care sites aimed at promoting person-centredness of integrated health and social care for older people living at home, and to gain insight into patterns in the experiences of multiple actors with these activities. This study shows that despite the variation between integrated care sites, similar activities have been implemented to promote person-centredness, which could be clustered into four categories: design of health and social care delivery process, staff training, communication and information exchange, and involvement in care and support. Overall, professionals and managers were satisfied with the implemented activities and thought that most activities positively influenced person-centredness. However, the experiences of older people and informal carers were mixed. For certain activities, for instance enablement services, an apparent discrepancy was identified between managers’ and professionals’ views on person-centred approaches compared with those of older people and informal carers.

### Understanding these results in the context of the literature

This study describes a range of different activities that aimed to enhance person-centredness and were undertaken in diverse settings across Europe. Many of the activities, such as co-designing care plans, sharing information about available services, and engaging close relatives in the care process, were also found in other existing studies about implementing and improving person-centredness [18,45,46,47]. However, this study also identified activities that were not directly related to person-centredness as conceptualised in the literature [19], but were nonetheless undertaken within the SUSTAIN case studies as part of a move towards person-centred care. These included, for example, staff training on awareness of older people’s health conditions and diseases, or training on inter-professional communication and collaboration. Such activities were implemented in the hope that they would improve person-centredness indirectly, and alongside other aspects of integrated care (e.g. prevention-orientation, safety, efficiency or coordination of care). This study therefore shows that different (types of) activities to embed and improve person-centredness in integrated care do co-exist. As also stressed in earlier studies [45,48], the operationalisation of person-centred care takes many different shapes and forms. In this study, such differences included variations in local priorities, and differences in the receptivity and readiness of older people to receive adapted, person-centred services, due to local cultural and historical factors. Since improvement processes are highly context-dependent, a one-size-fits-all approach to person-centred care would probably have been inappropriate in any case [21,49].

This study demonstrates that the integrated care sites had taken steps to embed and improve person-centred care, by implementing activities that aimed to improve several elements of person-centredness as conceptualised in the literature [19]. However, this study also demonstrates that further enhancements towards a person-centred care approach were still required. Some elements of person-centredness were underrepresented or posed mixed experiences between service providers and service users. As in previous studies [16,23,50], challenges were experienced in delivering supported self-care and shared decision-making, which highlight difficulties in empowering older people. Instead of considering service users as experts in their own care and support, and making them full and essential members of their care team, empowerment is often reduced to ‘patient education’ [50,51]. It seemed that some health and social care professionals remained paternalistic in their approaches to care and support and did not fully prioritise the meaningful engagement and empowerment of older people [22]. Barriers reported from the provider perspective included time and resource restrictions, professionals’ competence gaps, or preconceptions about which older people or clinical situations would benefit from active involvement or shared decision-making [19,52,53].

However, it should also be noted that activities that aimed at promoting person-centredness – including active participation, empowerment and leadership from older people – may not always match older people’s own preferences and capabilities [54,55]. For older people, elements such as a trusting and accommodating care relationship, empathetic communication and continuity in providers may be more important aspects of person-centred care than their active participation [56,57]. Furthermore, barriers to greater participation in the decision-making process, such as communication issues and cognitive impairment, are more prominent among older people [58]. For older people experiencing difficulties securing involvement in their care and support or navigating complex health and social care systems, receiving support to get the right care at the right time, such as in care advocacy initiatives, can be helpful [59,60]. Overall, older people’s individual interests and preferences regarding the role they wish or are able to play are important, and should be supported and respected when striving for meaningful and purposeful engagement of older people [56,57,61].

This study highlighted the difference in views and interpretations of person-centred care between service providers and service users (i.e. older people and their informal carers). This finding has also been observed in other studies [28,62,63]. Several methodological, clinical and contextual factors contributing to these different views have been described [28]. One of the potential explanations is the limited involvement of service users, not only in their own care and support, but also in the more extensive processes of service development and improvement [60,64]. Despite increasing recognition of the distinctive role service users may play in those latter processes, successful involvement remains limited [65,66]. Also, we found little representation and engagement of older people and informal carers in the SUSTAIN improvement processes, with only a few sites including representatives from patient advocacy organisations, despite the literature recommending the active involvement of service users (or their representative organisations) in successful improvement processes [67,68]. Their experiences and insights had been found to help identify potential improvements not previously identified by professionals [50,64,67]. As a result of their inputs, improvements were more likely to respect service users’ values and beliefs, thereby incorporating aspects of improvement important to them. Crucial components for promoting involvement include clarity about the rationale for their involvement and clarity about their roles and responsibilities [67].

### Methodological considerations

This paper describes a multiple embedded case study, in which individual case studies were compared and findings integrated. The individual case studies provided the opportunity to understand the coherence between individual data sources and the local context of an integrated care site. However, comparing and integrating the individual case studies across diverse countries and contexts posed potential methodological challenges. First, differences in the professional and cultural backgrounds of SUSTAIN’s research partners, who worked across different (political, economic and historical) contexts, may have influenced their interpretations of person-centredness. This diversity, in turn, may have contributed to small differences in case study descriptions. Second, the SUSTAIN project involved seven European countries. Therefore, data were collected in multiple languages. Some countries even have multiple (official) languages, such as Estonia, where both the Estonian and Russian languages are spoken. This complicated uniform and standardised measurement and comparison between case studies, particularly with regard to the qualitative data sources.

To address these challenges, a lot of effort was put into achieving harmonisation and alignment between case studies. Regular and structured discussions among the project partners enabled the development and use of standardised tools and procedures for data collection and analyses as well as for building the case studies. This supported comparability and comparison between individual case studies. Furthermore, research partners coordinating the overarching analysis supported local research partners in step 2 and step 3 of building the individual case studies (Table 3) to review and support internal consistency. No inconsistencies between research partners were found, which provides a reasonable degree of confidence about the consistency of approach across the case studies. We were therefore able to conduct a reliable overarching analysis of findings from individual case studies in different countries and contexts.

In this study, a case study design was adopted because of its perceived potential to evaluate complex community-based activities that were context bound, noted for their differences in implementation, and serving multiple purposes simultaneously [29]. While this type of design did not allow us to draw conclusions about the effectiveness of the various sites’ activities, we were able to provide insights into the experiences and perceptions of stakeholders from different evidence sources and their various viewpoints. New approaches, such as the case studies that were employed in SUSTAIN, are increasingly being recognised as helpful and are therefore recommended for the evaluation of complex community-based interventions. Furthermore, it should be noted that despite the fact that much effort has been put into uncovering older people’s experiences of service use, it has continued to be difficult to capture data about the features of care and support that really mattered to them [69]. Due to the data collection tools that were used, SUSTAIN’s research partners and local care staff also found it challenging to document the experiences of older people with multiple health and social care needs. It is recommended future research should specifically focus on how to overcome such problems.

### Conclusion

This study shows that stakeholders from integrated care sites across Europe undertook a wide variety of efforts to place older people at the centre of their care and support. Experiences from multiple perspectives showed that several of the activities undertaken have the potential to promote person-centredness. However, not all of the efforts were successful or generated the intended consequences for older people. Further improvements in integrated service design and delivery are required to engage and empower older people more widely and more effectively in their care and support. Such improvements should be critically evaluated from the separate perspectives of users, their friends and families, and also those responsible for their planning and allocation. In addition, the meaningful involvement of older people in improvement projects is a fundamental prerequisite if they are to be more fully person-centred – and, thus, increasingly tailored to their needs and preferences for integrated care and support.

## Additional Files

The additional files for this article can be found as follows:

10.5334/ijic.5427.s1 Appendix 1. Analysis templates for each data source.

10.5334/ijic.5427.s2 Appendix 2. Proposition analysis framework for site-specific overarching analysis.
