# Supplementary material for: Improving Person-Centredness in Integrated Care for Older People: Experiences from Thirteen Integrated Care Sites in Europe
Source: Int J Integr Care. 2020 Jun 26;20(2):16. doi: 10.5334/ijic.5427 (PMC7319083; doi:10.5334/ijic.5427)
Supplement: Appendix 1. — Analysis templates for each data source. [file ijic-20-2-5427-s1.pdf]

Appendix 1: Analysis templates for each data source

Analysis template demographic data user

Instructions:

1. Create a table (Table 1) showing the characteristics of the users in your 7-11 months sample. This information can be retrieved from the output file of SPSS/SAS (or any other statistical program). Please note that thematic statements no longer have to be formulated for the quantitative data since they are only used in qualitative research. Instead, please analyze your quantitative data and put the outcomes in the tables suggested in this document. Please write a short summary sheet of the most striking findings, including your interpretation of the findings.
2. Repeat step 1 for the data from your user sample at the 13-17 months assessment.
3. Write a summary report reflecting most striking differences in the characteristics of your 7-11 and 13-17 months user samples.

Table 1. Demographic data of users.

**Characteristic**	**Subgroup**	**N(%) or mean (SD)**
Sex	*Male*	
	Female	
Age group	*65-74 years*	
	75-84 years	
	85+ years	
Education level	*Low*	
	Middle	
	High	
Marital status	*Married/cohabiting*	
	Separated	
	Divorced	
	Widowed	
	Single	
Living situation	*Living at home alone*	
	Living at home with your spouse/partner	
	Living at home with at least one other family member	
	Living at home alone with at least one roommate	
	Other	
Medical conditions	***Number of conditions***	
	Anxiety/panic disorder	
	Asthma, chronic bronchitis, lung emphysema or COPD	
	Broken hip	
	Broken bones other than a broken hip	
	Cancer	
	Dementia/Alzheimer's	
	Depression	
	Diabetes	
	Dizziness with falling	
	Hearing problems	
	Heart failure	
	Involuntary loss of urine (incontinence)	
	Loss of bone tissue (osteoporosis)	
	Persistent back pain	
	Problems with vision	
	Prostate symptoms caused by benign prostate enlargement	
	Stroke, cerebral haemorrhage, cerebral infarction or transient ischemic attack (TIA)	
	Wearing of the joints (arthrosis, degenerative arthritis) of hips or knees	
	Any other condition	

Characteristic	Subgroup	N(%) or mean (SD)
	Prefer not to say	

Summary report
- Our sample included xx users. About xx% of them were females.
- Most users were in the age 75-84 years
- The average number of medical conditions was xx. Most prevalent conditions were...
- Etc.

Analysis template demographic data carer

Instructions:
1. Create a table (Table 1) showing the characteristics of the carers in your 7-11 months sample. This information can be retrieved from the output file of SPSS/SAS (or any other statistical program). Please note that thematic statements no longer have to be formulated for the quantitative data since they are only used in qualitative research. Instead, please analyze your quantitative data and put the outcomes in the tables suggested in this document. Please write a short summary sheet of the most striking findings, including your interpretation of the findings.
2. Repeat step 1 for the data from your carer sample at the 13-17 months assessment.
3. Write a summary report reflecting most striking differences in the characteristics of your 7-11 and 13-17 months carer samples.

Table 1. Demographic data of carers.

**Characteristic**	**Subgroup**	**N(%) or mean (SD)**
Sex	*Male*	
	Female	
Age group	*18-24 years*	
	25-34 years	
	35-44 years	
	45-54 years	
	55-64 years	
	65-74 years	
	75-84 years	
	85+ years	
Education level	*Low*	
	Middle	
	High	
Marital status	*Married/cohabiting*	
	Separated	
	Divorced	
	Widowed	
	Single	
Relationship to user	*Spouse/partner*	
	Son	
	Son-in-law	
	Daughter	
	Daughter-in-law	
	Other family member/relative	
	Hired carer (paid for by the user/user's family)	
	Hired carer (paid for by the state or other insurance)	
	Other	
Living situation	*With user*	
	Close-by the user (within 5 km)	
	Further away from the user (more than 5 km)	
Paid job	*Yes*	
	No	
	Number of working hours per week	
Caregiving	*Number of hours per week spent on caregiving*	
Needs assessment	*Yes*	
	No	
Care plan	*Yes*	
	No	

Summary report
- Our sample included xx carers. About xx% of them were females.
- Most users were in the age 55-64 years.
- Most of the carers were either daughters or daughters in law.
- None of the carers had their own care plan.
- Etc.

Analysis template demographic data professional

Instructions:

1. Create a table (Table 1) showing the characteristics of the professionals in your 6 months sample. This information can be retrieved from the output file of SPSS/SAS (or any other statistical program). Please note that thematic statements no longer have to be formulated for the quantitative data since they are only used in qualitative research. Instead, please analyze your quantitative data and put the outcomes in the tables suggested in this document. Please write a short summary sheet of the most striking findings, including your interpretation of the findings.
2. Repeat step 1 for the data from your professionals sample at the 18 months assessment.
3. Write a summary report reflecting most striking differences in the characteristics of your 6 and 18 months professionals samples.

Table 1. Demographic data of professionals.

**Characteristic**	**Subgroup**	**N(%)**
Sex	*Male*	
	Female	
Age group	*18-24 years*	
	25-34 years	
	35-44 years	
	45-54 years	
	55-64 years	
	65+ years	
Nationality	*Please specify based on your sample*	
Education level	*Low*	
	Middle	
	High	
Occupation	*Please specify based on your sample*	
Staff group	*Administrative & clerical*	
	Allied health professionals	
	Medical & dental	
	Nursing	
	Social and community workers/social work	
	Other	
Employment status	*Employee (permanent contract)*	
	Employee (temporary contract)	
	Agency worker	
	Self-employed	
	Locum	
	Student	
	Volunteer	
	Other, please specify	
Working hours	*Full time*	
	Part time	
Area of work/care setting	*Health care organisation (acute hospital)*	
	Health care organisation (primary care, community neighbourhoods/patches, community hospital)	
	Health and social care—integrated organisation	
	Social care/local government	
	Other	
Percentage of colleagues that are men/women	*Male*	
	Female	

Summary report

- Our sample included xx professionals. About xx% of them were females.

- Most professionals were in the age of 45-54 years.
- Most of the professionals were employed part time and worked for either a healthcare organisation or a social care organisation. Other settings that were mentioned included the voluntary sector etc.
- Etc.

Analysis template demographic data manager

Instructions:

1. Create a table (Table 1) showing the characteristics of the managers in your 6 months sample. This information can be retrieved from the output file of SPSS/SAS (or any other statistical program). Please note that thematic statements no longer have to be formulated for the quantitative data since they are only used in qualitative research. Instead, please analyze your quantitative data and put the outcomes in the tables suggested in this document. Please write a short summary sheet of the most striking findings, including your interpretation of the findings.
2. Repeat step 1 for the data from your managers sample at the 18 months assessment.
3. Write a summary report reflecting most striking differences in the characteristics of your 6 and 18 months managers samples.

Table 1. Demographic data of managers.

**Characteristic**	**Subgroup**	**N(%)**
Sex	*Male*	
	Female	
Age group	*18-24 years*	
	25-34 years	
	35-44 years	
	45-54 years	
	55-64 years	
	65+ years	
Nationality	*Please specify based on your sample*	
Education level	*Low*	
	Middle	
	High	
Occupation	*Please specify based on your sample*	
Area of work/care setting	*Health care organisation (acute hospital)*	
	Health care organisation (primary care, community neighbourhoods/patches, community hospital)	
	Health and social care—integrated organisation	
	Social care/local government	
	Other	
Employment status	*Employee (permanent contract)*	
	Employee (temporary contract)	
	Agency worker	
	Self-employed	
	Interim	
	Other, please specify	
Working hours	*Full time*	
	Part time	
Average percentage of colleagues that are men/women	*Male*	
	Female	

Summary report

- Our sample included xx managers. About xx% of them were females.
- Most managers were in the age of 45-54 years.
- Most of the managers were employed fulltime and worked for either a healthcare organisation or a social care organisation.
- Etc.

Analysis template P3CEQ

Quantitative data

Instructions:

1. Create a table (Table 1) showing the caregivers involved in the care processes of the older people in your sample. This information can be retrieved from the output file of SPSS/SAS (or any other statistical program). Please note that thematic statements no longer have to be formulated for the quantitative data since they are only used in qualitative research. Instead, please analyze your quantitative data and put the outcomes in the tables/templates suggested in this document. Please write a short summary sheet of the most striking findings, including your interpretation of the findings.
2. Create a table (Table 2) presenting the average score per single item of the P3CEQ 7-11 months assessment. Please write a short summary sheet of the most striking findings, including your interpretation of the findings.
3. Subgroup analyses will be conducted for the overarching analyses only and not for the site-specific analyses due to small sample sizes. However, your sites may be interested in differences between groups. So we are suggesting that, where there are sufficient data, partners can give the steering groups some impressions or ‘subgroup commentary’ as feedback using Table 3. Most striking findings can be included in the short summary sheet (see above). Subgroups you may include: sex, age group, educational level and living situation.
4. Repeat step 1-2 for the data from the P3CEQ 13-17 months assessment.
5. Write a summary report reflecting most striking differences in percentages/scores between the 7-11 and 13-17 months assessment data (e.g. The overall mean P3CEQ score was similar at 7-11 and 13-17 months assessment, implying that experiences with person-centred care did not change over time; Largest changes were found on items 3 and 6 of the P3CEQ, reflecting that, at 13-17 months assessment older people were more considered as a ‘whole’ person and found their care more joined up as compared to 7-11 months assessment).

Table 1. Caregivers involved in care process of older person.

**Type of caregiver**	**% (N=)**
GP	100% (n=15)
Social services	13% (n=2)
Hospital as an inpatient	Etc.
Allied health services (e.g. physiotherapy)	
Voluntary services (e.g. Age UK)	
Nurse (community, practice)	
Mental health services	
Hospital as an outpatient	
Agency support services (e.g. care services)	
Others	

Summary report

- The P3CEQ was completed by xx older people.
- All older people indicated that the GP was involved in their care process.
- Social services were less involved. Only 13% of the older people indicated that social care professionals were involved in their care.
- Etc.

Table 2. P3CEQ scores; individual items, subscales and overall score.

		% (N=)	Mean score (SD)	% yes (N=)
**Individual P3CEQ items**	*1. Most important in managing health and wellbeing*	100% (n=15)	2.3 (0.5)	-
	2. Older person involved in decision-making as much as wanted	Etc.		-
	3. Considered as a 'whole' person			-
	4. Necessary to repeat information			-
	5. Extent to which care is joined up			-
	6. Availability coordinating professional(s)			-
	7a. Availability of care plan<br>*7b. Care plan available to older person*<br>*7c. Extent to which care plan is useful for older person*<br>*7d. Extent to which professionals follow the same care plan*			-
	8. Extent to which received support is sufficient to older person			-
	9. Extent to which information when needed is useful			-
	10. Confidence in managing health and wellbeing			-
	11a. Wish to involve family and friends in decision-making		-	40% (n=6)
	11b. Family/ friends involved in decision-making as much as wanted			-
**Total P3CEQ score (0-30)**	*Total score*			-

Summary report
- The P3CEQ was completed by xx respondents.
- The overall P3CEQ mean score was 28, implying that reflecting that older people had good experiences with person-centred care.
- The mean score on item 7b was relatively low as compared to the other items.
- Etc.

Table 3. Mean P3CEQ scores for the different subgroups.

	**Subgroups**									
	**Sex**		**Age group**			**Living situation**		**Educational level**		
	Males (mean; SD)	*Females (mean; SD)*	*Age group 65-74 years (mean; SD)*	*Age group 75-84 years (mean; SD)*	*Age group > 85 years (mean; SD)*	*Living alone (mean; SD)*	*Living together (mean; SD)*	*Low (mean; SD)*	*Middle (mean; SD)*	*High (mean; SD)*
**Total P3CEQ score (0-30)**										

Summary report

- We distinguished the following subgroups: sex, age group, living situation and educational level.
- The mean total P3CEQ score was higher for males than for females.
- Etc.

Qualitative data

Instructions:

1. Analyse the qualitative data from the 7-11 months measurement by summarizing most striking answers and comments. Also for these data, thematic statements will no longer be needed. Please use Table 4 to group the findings to create a logical flow with the questionnaire.
2. Repeat step 1 for the qualitative data from the 13-17 months measurement.
3. Write a summary report reflecting most striking differences between 7-11 and 13-17 months assessment data, if relevant/possible.

Table 4. Qualitative data collected with the P3CEQ.

Interview Domain	Findings from all completed P3CEQ surveys
**Involvement in care**<br>*Comments related to items 1-3*	
**Coordinated care**<br>*Comments related to items 4-6*	
**Care planning**<br>*Comments related to items 7a-d*	
**Self management**<br>*Comments related to items 8-10*	
**Family involvement**<br>*Comments related to items 11ab*	
**Improvements to care**<br>*Open question*	
**Other topics not covered by schedule**<br>*Open question*	

Summary report

- Although older people indicated to not have access to their care plans, they also indicated that they didn't care. They were not aware of having a care plan and were not interested in the information that was written in it.
- Several respondents indicated to have difficulties with answering questions xx and xx. They indicated that xxx. Therefore the percentage of missing data for these questions is relatively large.

Analysis template PCHC

Instructions:

1. Create a table (Table 1) presenting the average score per single item of the PCHC 7-11 months assessment. This information can be retrieved from the output file of SPSS/SAS (or any other statistical program). Please note that thematic statements no longer have to be formulated for the quantitative data since they are only used in qualitative research. Instead, please analyze your quantitative data and put the outcomes in the tables/templates suggested in this document. Please write a short summary sheet of the most striking findings, including your interpretation of the findings and a summary of any comments .
2. Subgroup analyses will be conducted for the overarching analyses only and not for the site-specific analyses due to small sample sizes. However, your sites may be interested in differences between groups. So we are suggesting that, where there are sufficient data, partners can give the steering groups some impressions or 'subgroup commentary' as feedback using Table 3. Most striking findings can also be included in the short summary sheet (see above). Subgroups you can think of include: sex, age group, educational level and living situation.
3. Repeat step 1-2 for the data from the PCHC 13-17 months assessment.
4. Write a summary report reflecting most striking differences in percentages/scores between the 7-11 and 13-17 months assessment data.

Table 1. *PCHC scores; individual items, subscales and overall score.*

		**N** ***(sample size)***	**% of the total sample**	**Mean score (SD)**
**Scores on Part A- Individual PCHC items (1-10)**	*1. Keeping control of health care*	15	-	7.8 (0.6)
	2. Getting enough support from people close to me	13	-	
	3. Person who controls care<br>*Myself*<br>*My family/relatives/friends/neighbours*<br>*Myself and family/relatives/friends/neighbours*<br>*Someone else*	14	 70%<br>20%<br>5%<br>5%	-
	4. Importance to stay in control	etc.	-	
**Scores on Part B - Individual PCHC items (1-5)**	*5. Knowing when it is time to call on professional care*		-	3.5 (0.3)
	6. Ability to find information about health or care		-	
	7. Ability to find out aids or services		-	
	8. Knowing where to apply for care, aids or services		-	
	9. Ability to arrange care, aids or services		-	
	10. Understanding health care organisations		-	
	11. Managing to get to healthcare professionals		-	
	12. Ability to keep track of appointments		-	
	13. Ability to explain what is going on		-	
	14. Ability to ask questions about health or treatment		-	
	15. Informing professionals about wishes		-	
	16. Standing up for myself		-	
	17. Dealing with medication<br>*Not applicable*		 %	

	18. Carying out advice<br>*Not applicable*		%	
	19. Doing what is necessary		-	
	20. Adapting to setbacks		-	
	21. Knowing when to arrange complex care		-	
	22. When I need complex care, taking part in decisions		-	
	23. When I need complex care, coping financially		-	
	24. When mind deteriorates, taking necessary preparations		-	
	25. When needing help in house, counting on people		-	
	26. Get professional care, counting on people		-	
	27. When in emergency, counting on a plan		-	
	28. Asking people for help, when needed<br>*Not applicable*		%	
	29. When being helped, taking part in decisions<br>*Not applicable*		%	
**PCHC subscale scores[1]**	*1. Perceived personal control in health care (items 5-15, 17-18)*		-	
	2. Anticipated personal control in health care (items 21-23)		-	
	3. Perceived support from social network (items 25-26, 28)		-	
**Mean PCHC score, based on part B items (1-5)**	*Total score*		-	3.2 (0.5)

1. Items 16, 19, 20, 24, 27 and 29 have been excluded from the factor structure for several reasons, see Claassens et al. 2016.

Summary report
- The PCHC was completed by xx respondents.
- Average scores on the different items varied from xx to xx, showing that xxx.
- Almost all respondents (95%) indicated that they were the ones that were in control of their care.
- Percentage of missing values was particularly high for items xx and xx. Potential explanations may be that xxx.
- Scores on the three subscales were xxx.
- The overall mean score of the items of part B of the survey was 3.2.

Table 2. Overall mean PCHC score for the different subgroups.

	**Subgroups**									
	**Sex**		**Age group**			**Living situation**		**Educational level**		
	Males (mean; SD)	*Females (mean; SD)*	*Age group 65-74 years (mean; SD)*	*Age group 75-84 years (mean; SD)*	*Age group > 85 years (mean; SD)*	*Living alone (mean; SD)*	*Living together (mean; SD)*	*Low (mean; SD)*	*Middle (mean; SD)*	*High (mean; SD)*
**Overall mean PCHC score - part B (1-5)**										

Summary report

- We distinguished the following subgroups: sex, age group, living situation and educational level.
- The overall mean PCHC mean score was higher for males than for females.
- Etc.

Analysis template TCI 14 items

Instructions:

1. Create a table (Table 1) presenting the average score per single item of the TCI 0-6 months assessment. This information can be retrieved from the output file of SPSS/SAS (or any other statistical program). Please note that thematic statements no longer have to be formulated for the quantitative data since they are only used in qualitative research. Instead, please analyze your quantitative data and put the outcomes in the tables/templates suggested in this document. Please write a short summary sheet of the most striking findings, including your interpretation of the findings and including a summary of any comments that respondents gave during the completion the TCI.
2. Subgroup analyses will be conducted for the overarching analyses only and not for the site-specific analyses due to small sample sizes. However, your sites may be interested in differences between groups. So we are suggesting that, where there are sufficient data, partners can give the steering groups some impressions or 'subgroup commentary' as feedback using Table 2. Most striking findings can also be included in the short summary sheet (see above). Subgroups you can think of include: sex, area of work and type of profession (manager vs. professional).
3. Repeat step 1-2 for the data from the TCI at 13-17 months.
4. Write a summary report reflecting most striking differences in scores on individual TCI items, the TCI subscales and overall TCI score between the 0-6 months and 13-17 months assessment data (e.g. The overall mean TCI score was a bit higher at 13-17 months assessment as compared to 0-6 months, implying that respondents' experience with team coherence improved over time, etc.).

Table 1. TCI scores; individual items, subscales and overall score.

		**% (N=)**	**Mean score (SD)**
**Scores on individual TCI items (1-5)**	*1. Agreement with objectives*	100% (n=12)	4.1 (0.4)
	2. Team's objectives clearly understood	67% (n=8)	
	3. Team's objectives achievable	etc.	etc.
	4. Worth of the objectives to the organisation		
	5. 'We are together' attitude		
	6. People keep each other informed		
	7. People feel understood and accepted		
	8. Real attempts to share information		
	9. Preparedness to basic questions		
	10. Critical appraisal of weaknesses		
	11. Building on each other's ideas		
	12. Search for new ways of looking at problems		
	13. Time taken to develop ideas		
	14. Cooperation in developing and applying ideas		
**Scores on TCI subscales**	*Vision (items 1, 2, 3, 4)*		
	Participative safety (items 5, 6, 7, 8)		
	Task orientation (items 9, 10, 11)		

		% (N=)	Mean score (SD)
	Support for innovation (items 12, 13, 14)		
**Overall TCI mean score (1-5)**	*Total score*		

Summary report
- The TCI was completed by xx respondents.
- The overall TCI mean score was xx, implying that xxx.
- The average scores on the different individual items varied from xx to xx. Percentage of missing data was largest for items xx and xx. This might have been related to the fact that xxx.
- Average score on TCI subscale xx was higher as compared to the other three subscales. This seems to reflect that xxx.
- Etc.

Table 2. Mean TCI scores for different subgroups.

	**Subgroups**								
	**Sex**		**Area of work**					**Type of profession**	
	Males (mean; SD)	*Females (mean; SD)*	*Hospital (mean; SD)*	*Primary care organisation (mean; SD)*	*Integrated care organisation (mean; SD)*	*Social care organisation (mean; SD)*	*Other(mean; SD)*	*Professionals (mean; SD)*	*Managers (mean; SD)*
**TCI total mean score (1-5)**									

Summary report
- We distinguished the following subgroups: sex, area of work and type of profession.
- Total TCI mean score was higher for males than for females
- Etc.

Analysis template user interview

Instructions:
1. This template should be used to extract and analyse data from the user interviews.
2. This process can be started as soon as the user interviews have been conducted and transcribed.
3. Code all data based on the domains outlined in the table below. You can either paste extracts into the table (in the 'coded data from all user interviews' column) and analyse using Word, or use a program for analysing qualitative data. Data that are entered into the 'coded data' section of table can be in your own language.
4. For all extracts or quotes, identify where it is from, using the respondent code (e.g. SP-1-U-001-C) in brackets.
5. After extracting coded data from all the user interview transcripts, formulate thematic statements for each of the domains. For all thematic statements, mention the data source, in this case UI = User Interview. While the coded data can be in your own language, the thematic statements (together with some quotes) need to be translated into English. It is important to remember that you will be generating thematic statements based on a number of people saying similar things, not just one or two, however powerful their statement. So if one person only has raised an issue in one of the subthemes, it cannot be developed into a thematic statement unless it is echoed by others during the interviews.
6. Enter onto the online database a) your thematic statements in English, some selected quotes and b) the final version of your completed analysis template and c) your interview transcripts.

Interview domain	Coded data from all user interviews	Thematic statements
**Support at home and description of support**	Nb. Mention the respondent code in brackets after each quotation, for example: (SP-1-U-001-C)	Nb. Mention the data source in brackets after each thematic statement, in this case: (UI)<br><br>Please note: only one subject/topic per statement)
**Perception of Initiative** (*length of time receiving service, who comes to the home, other types of contact, thoughts on amount of time with workers, how person is treated*)		
**Co-ordination** (*knowledge of services, how workers work together, who*		

and how to contact people, nature of this contact)		
**Perception of person-centredness** (*meeting and prioritising needs, maximising independence, involvement in setting goals/care planning, participation in care, achieving goals, understanding information and being listened to, gender issues*)		
**Prevention-orientation** (*help to live independently, planning ahead, coping in ill health*)		
**Safety consciousness** (*confidence and managing at home, addressing safety in care plan, medication review, type and usefulness of equipment*)		
**Comparing services** (*how previous service compared to current one on above topics*)		
**Finances** (*how care is paid for, control of healthcare spending, adequacy of money to cover costs, how shortfalls are paid, availability of expert advice*)		
**Other topics not covered by schedule**		

Analysis template carer interview

Instructions:

1. This template should be used to extract and analyse data from the carer interviews.
2. This process can be started as soon as the carer interviews have been conducted and transcribed.
3. Code all data based on the domains outlined in the table below. You can either paste extracts into the table (in the 'coded data from all carer interviews' column) and analyse using Word, or use a program for analysing qualitative data. Data that are entered into the 'coded data' section of table can be in your own language.
4. For all extracts or quotes, identify where it is from, using the respondent code (e.g. SP-1-C-001-C) in brackets.
5. After extracting coded data from all the carer interview transcripts, formulate thematic statements for each of the domains. For all thematic statements, mention the data source, in this case CI = Carer Interview. While the coded data can be in your own language, the thematic statements (together with some quotes) need to be translated into English. It is important to remember that you will be generating thematic statements based on a number of people saying similar things, not just one or two, however powerful their statement. So if one person only has raised an issue in one of the subthemes, it cannot be developed into a thematic statement unless it is echoed by others during the interviews.
6. Enter onto the online database a) your thematic statements in English, some selected quotes and b) the final version of your completed analysis template and c) your interview transcripts.

Interview domain	Coded data from all carer interviews	Thematic statements
**Support at home and description of support**	Nb. Mention the respondent code in brackets after each quotation, for example: (SP-1-C-001-C)	Nb. Mention the data source in brackets after each thematic statement, in this case: (CI)
**Perception of Initiative** (*contact with workers, thoughts on amount of time with workers, how user and carer are treated, trust*)		
**Co-ordination** (*knowledge of services, how workers work together, who and how to contact people, nature of this contact*)		

**Perception of person-centredness**<br>(*involvement in setting goals/care planning of user, maximising independence of user, meeting needs of carer, involvement in setting goals/care planning of carer, support for carer, understanding information and being listened to*)		
**Prevention-orientation**<br>(*help independent living of user, information keep living at home, offered training in practical care, coping in ill health*)		
**Safety consciousness**<br>(*confidence and managing at home, addressing safety in care plan, care and medication review, type and usefulness of equipment*)		
**Comparing services**<br>(*how previous service compared to current one on above topics*)		
**Finances**<br>(*financial burden carer*)		
**Other topics not covered by schedule**		

Analysis template dyad interview

Instructions:

1. This template should be used to extract and analyse data from the dyad interviews.
2. This process can be started as soon as the dyad interviews have been conducted and transcribed.
3. Code all data based on the domains outlined in the table below. You can either paste extracts into the table (in the 'coded data from all dyad interviews' column) and analyse using Word, or use a program for analysing qualitative data. Data that are entered into the 'coded data' section of table can be in your own language.
4. For all extracts or quotes, identify where it is from, using the respondent code (e.g. SP-1-U-001-C or SP-1-C-001-C) in brackets.
5. After extracting coded data from all the dyad interview transcripts, formulate thematic statements for each of the domains. For all thematic statements, mention the data source, in this case DI = Dyad Interview. While the coded data can be in your own language, the thematic statements (together with some quotes) need to be translated into English. It is important to remember that you will be generating thematic statements based on a number of people saying similar things, not just one or two, however powerful their statement. So if one person only has raised an issue in one of the subthemes, it cannot be developed into a thematic statement unless it is echoed by others during the interviews.
6. Enter onto the online database a) your thematic statements in English, some selected quotes and b) the final version of your completed analysis template and c) your interview transcripts.

Interview domain	Coded data from all dyad interviews	Thematic statements
**Support at home and description of support**	Nb. Mention the respondent code in brackets after each quotation, for example: (SP-1-U-001-C or SP-1-C-001-C)	Nb. Mention the data source in brackets after each thematic statement, in this case: (DI)
**Perception of Initiative** (*length of time receiving service, who comes to the home, other types of contact, thoughts on amount of time with workers, how user and carer are treated, carer contact with workers, trust*)		
**Co-ordination** (*knowledge of services, how workers work together, who*		

and how to contact people, nature of this contact)		
**Perception of person-centredness**<br>(*meeting and prioritising needs of user, maximising independence of user, involvement of user and carer in setting goals/care planning of user, participation in care for user, achieving goals of user, gender issues, meeting needs of carer, involvement in setting goals/care planning of carer, support for carer, understanding information and being listened to*)		
**Prevention-orientation**<br>(*help to live independently, planning ahead, coping in ill health, information keep living at home, offered training in practical care*)		
**Safety consciousness**<br>(*confidence and managing at home, addressing safety in care plan, medication review, type and usefulness of equipment*)		
**Comparing services**<br>(*how previous service compared to current one on above topics*)		
**Finances**<br>(*how care is paid for, control of healthcare spending, adequacy*		

of money to cover costs, how shortfalls are paid, availability of expert advice, financial burden carer)		
**Other topics not covered by schedule**		

Analysis template professional focus group

Instructions:

1. This template should be used to extract and analyse data from the professional focus groups.
2. This process can be started as soon as the focus groups have been conducted and transcribed.
3. Code all data based on the domains outlined in the table below. You can either paste extracts into the table (in the 'coded data from all professional focus groups' column) and analyse using Word, or use a program for analysing qualitative data. Data that are entered into the 'coded data' section of table can be in your own language.
4. For all extracts or quotes, identify where it is from, using the respondent code (e.g. SP-1-P-001-C) in brackets.
5. After extracting coded data from all the focus group transcripts, formulate thematic statements for each of the domains. For all thematic statements, mention the data source, in this case PFG=Professional Focus Group. While the coded data can be in your own language, the thematic statements (together with some quotes) need to be translated into English. It is important to remember that you will be generating thematic statements based on a number of people saying similar things, not just one or two, however powerful their statement. So if one person only has raised an issue in one of the subthemes, it cannot be developed into a thematic statement unless it is echoed by others during the interviews.
6. Enter onto the online database a) your thematic statements in English, some selected quotes and b) the final version of your completed analysis template and c) your interview transcripts.

Interview domain	Coded data from professional focus groups	Thematic statements
**Position and role in the improvement process**	Nb. Mention the respondent code in brackets after each quotation, for example: (SP-1-P-001-C)	Nb. Mention the data source in brackets after each thematic statement, in this case: (PFG)
**Implementation improvement project** *(improvement project carried out as in original plan, deviations from original plan)*		
**Contextual issues** *(aspects of improvement plan implemented successfully and less successfully, pivotal role of collaborating organisation/colleague, influence of governance*		

Interview domain	Coded data from professional focus groups	Thematic statements
arrangements, leadership, accountability, policy issues, organisational issues, collaboration, interpersonal relations, availability of resources, financial issues)		
**Outcomes improvement project** *(experiences improvement process, achieving goals as in plan, effects of improvement project on integration, person-centredness, prevention-orientation, safety and efficiency)*		
**Reflection on project** *(most important facilitators and barriers, effect of facilitators and barriers on outcomes, rate of change due to project, unexpected changes, needs for further improving services)*		
**Top three lessons** *(advice to others undertaking a similar improvement project)*		
**Other topics not covered by schedule**.		

Analysis template manager interview

Instructions:

1. This template should be used to extract and analyse data from the manager interviews.
2. This process can be started as soon as the manager interviews have been conducted and transcribed.
3. Code all data based on the domains outlined in the table below. You can either paste extracts into the table (in the 'coded data from all manager interviews' column) and analyse using Word, or use a program for analysing qualitative data. Data that are entered into the 'coded data' section of table can be in your own language.
4. For all extracts or quotes, identify where it is from, using the respondent code (e.g. SP-1-M-001-C) in brackets.
5. After extracting coded data from all the manager interview transcripts, formulate thematic statements for each of the domains. For all thematic statements, mention the data source, in this case MI = Manager Interview. While the coded data can be in your own language, the thematic statements (together with some quotes) need to be translated into English. It is important to remember that you will be generating thematic statements based on a number of people saying similar things, not just one or two, however powerful their statement. So if one person only has raised an issue in one of the subthemes, it cannot be developed into a thematic statement unless it is echoed by others during the interviews.
6. Enter onto the online database a) your thematic statements in English, some selected quotes and b) the final version of your completed analysis template and c) your interview transcripts.

Interview domain	Coded data from manager interview	Thematic statements
**Role in the improvement process**	Nb. Mention the respondent code in brackets after each quotation, for example: (SP-1-M-001-C)	Nb. Mention the data source in brackets after each thematic statement, in this case: (MI)
**Improvement project** *(improvement project carried out as in original plan, deviations from original plan)*		
**Contextual issues** *(aspects of improvement plan implemented successfully and less successfully, pivotal role of collaborating organisation/colleague, influence of governance arrangements, leadership, accountability, policy issues, organisational issues,*		

Interview domain	Coded data from manager interview	Thematic statements
collaboration, interpersonal relations, availability of resources, financial issues)		
**Outcomes improvement project** *(experience improvement process, achieving goals as in plan, effects of improvement project on integration, person-centredness, prevention-orientation, safety and efficiency)*		
**Reflection on project** *(most important facilitators and barriers, effect of facilitators and barriers on outcomes, unexpected changes, needs for further improving services)*		
**Top three lessons** *(advice to others undertaking a similar improvement project)*		
**Other topics not covered by schedule**.		

Analysis template Care Plan Template

Quantitative indicators

Instructions:

1. Create a table (Table 1) presenting per single item of the Care Plan Template in 7-11 months assessment whether or not the following has taken place during the user's time in the improved service. This information can be retrieved from the output file of SPSS/SAS (or any other statistical program). Please analyze your quantitative data and put the outcomes in the tables suggested in this document. Please write a short summary sheet of the most striking findings, including your interpretation of the findings.
2. Repeat for the data from the Care Plan Template 13-17 months assessment.
3. Write a summary report reflecting most striking differences between the two data collection periods.

Table 1. Quantitative indicators Care Plan Template

		Yes (%)	No (%)	Mean (SD)
**Part A - Core Quantitative Indicators**	*A needs assessment has taken place*	100%	0%	-
	The care plan describes which activities are actioned or being actioned	67%	33%	-
	The care plan is shared across different professionals	Etc.		-
	The care plan is shared across different organisations			-
	The user received a medication review			-
	The user received, or is receiving, advice on medication adherence			-
	The user received, or is receiving, advice on how to maintain independence (e.g. self-management)			-
	The user received, or is receiving, safety advice (e.g. security at home, prevention of falls) by staff			-
	Falls are being recorded in the care plan			-
	Number of emergency hospital admissions of the user	-	-	
	Length of stay per emergency admission of the user	-	-	
	Number of hospital readmissions of the user	-	-	

		Yes (%)	No (%)	Mean (SD)
**Part B – Additional Care Plan Indicators**	*The care plan contains user needs*			-
	The care plan contains goals the user wants to achieve			-
	The care plan describes which professional will do what to help the user achieving these goals			-
	The care plan contains roles of informal carers in relation to the goals			-
	The care plan describes what the user will do to achieve these goals			-
	The care plan describes which activities need to be done			-

Qualitative indicators

Instructions:

1. Analyse the qualitative data from the 7-11 months measurement by summarizing most striking findings (including where the data is obtained from). Also for these data, thematic statements will no longer be needed. Please use Table 2 to group the findings.
2. Repeat step 1 for the qualitative data from the 13-17 months measurement.
3. Write a summary report reflecting most striking differences between 7-11 and 13-17 months assessment data, if relevant/possible.

Table 2. Qualitative indicators Care Plan Template.

Care plan domain	Findings from the care plan data collection template (section 2)
**Goals**<br>(The goals or other outcomes described, the activities to achieve these goals or outcomes, the extent to which these goals or outcomes have been achieved)	
**Informal care providers (carers)**<br>(The roles and tasks of carers)	
**Staff**<br>(The roles and tasks of staff)	
**Shared care**<br>(How the care plan is shared across teams)	

**Medication adherence advice**<br>(How advice on medication adherence is received)	
**Maintaining independence**<br>(How advice on maintaining independence is received)	
**Safety advice**<br>(How advice on safety is received)	
**Needs assessment**<br>(who carried out the assessment, how was this done, any instruments used)	

Analysis template efficiency indicators: staff hours and costs of equipment and technology

Instructions:

1. Create a table (Table 1) presenting the number of staff hours of the total sample and for the 6-17 month period, the 6-11 month period and the 12-17 month period.
2. Please analyze your quantitative data and put the outcomes in the tables suggested in this document. Please write a short summary based on Table 1 of the most striking findings, most striking differences between the 6-11 month period and the 12-17 month period including your interpretation of the findings.
3. Create a table (Table 2) with the total costs for equipment and technology for implementing the improvement project in the national currency. Please write a short summary based on Table 2 of the most striking findings. Please use your national currency. For the purpose of the overarching analyses, these currencies will later be converted to euros by WP5.

Table 1. Mean number of additional staff hours per staff member per month

	**Number of staff**	**Total sample**	**Subgroups**	
	Mean number of staff that spent hours in implementing improvement project; mean number of professionals that…; mean number of managers that…	*Total number of (extra) hours for implementing the improvement project (of all staff)*	*Professionals: number of (extra) hours of professionals for implementing the improvement project*	*Managers: number of (extra) hours of managers for implementing the improvement project*
**Overall: Month 6-17**				
**Month 6-11**				
**Month 12-17**				

Summary report.

Short description of outcomes Table 1. What is striking when looking to the staff hours that were spent on implementing the improvement project? For example: how many changes in staff took place (staff turnover), how many and which areas of work (health care organization – acute hospital etc.) spent hours in implementing the improvement project, were hours mostly spent by one or two professionals or did staff spent about the same number of hours?

Difference between month 6-11 and month 12-17

Example: The total number of hours is a bit higher in month 6-11 as compared to month 12-17, indicating that staff members spent more extra hours on implementing the improvement project (working according to new agreements) between month 6-11 than in month 12-17.

Difference between professionals and manager(s)

Example: The number of hours is relatively higher for professionals as compared to the manager, indicating that professionals spent relatively more extra hours per month on implementing the improvement project (working according to new agreements) than the manager.

Table 2. Total costs of equipment and technology

**Specification of equipment and technology purchased for implementation of improvement project (e.g. costs IT, etc.)**	
**Total costs of equipment and technology (in national currency)**	

Summary report
- Costs for additional equipment and technology were xx.
- Costs were mostly related to new cell phones for the nurses and social workers.
Etc.

Analysis template – steering group and field notes

Instructions:

1. This template should be used to extract and analyse data from formal steering group meeting notes and your reflective notes on those meetings. You should also draw on additional data – field notes (for example correspondence with steering group members outside of meetings)
2. This process can be started as early as you like (different sites have different numbers of steering group meetings). **A summary analysis should be produced just before the month 12 and month 18** steering group to inform the discussion. A final analysis should be produced after the month 18 steering group.
3. Code all meeting notes based on the themes outlined in the table below. There is some guidance in italics in the sections to help you to think through what to enter, and examples drawn from a UK site in red. You can either paste extracts or write summary information into the table and analyse using Word, or use a program for analysing qualitative data. Data that are entered into the 'coded data' section of table can be in your own language.
4. For any extracts or summaries, **identify where it is from, using a code and date** (YY/MM): **SGM** for steering group minutes; **RN** for reflective notes; **FN** for field notes. E.g. SGM17/03; RN17/03; FN16/02
5. Please note that it is important not to 'invent' things just to fill the boxes. If there is no data in your steering group minutes, reflective notes or field notes that relates to a particular theme, just leave it blank.
6. Sometimes, an extract might fit in more than one box (where it overlaps two or more themes). In such cases, paste the extract in all themes that are relevant (this is like double or multiple coding).
7. After extracting coded data from ALL steering group meetings/notes, formulate thematic statements for each theme (examples are included in red). While the coded data can be in your own language, the thematic statements need to be translated into English. It is important to remember that you will be generating thematic statements based on a number of people saying similar things, not just one or two, however powerful their statement. So if one person only has raised an issue in one of the subthemes, it cannot be developed into a thematic statement unless it is echoed by others during the steering group meetings.
8. Enter onto the online database a) your thematic statements in English, and b) the final version of your completed analysis template and c) your minutes and field notes in your own language

GENERAL CONVENTIONS TO FOLLOW (as evidenced in the examples below):

i.) Make sure the year is included on every entry within the coded data boxes. You will then be able to consider time (and change over time) in the formulation of your thematic statements. E.g. "Lack of a dedicated leader at the start of the implementation period hindered the improvement process". "The introduction of a political imperative to free up more beds at month 7 helped to get strategic level support for the improvement project, which enabled new resources to be allocated to it".

ii.) To anonymise the data, use square brackets [like this] to replace names of people or organisations, as I have done in the example below.

Theme	Coded Data from All Steering Group Meeting Minutes / Notes	Thematic statements
**Governance arrangements in the improvement project**<br>- Composition and functioning of steering group<br>- Did any groups feed into the steering group?<br>- Did the steering group feed into other groups?	*Take a look at regularity of meetings, attendance, composition of group, etc and insert key information here (SGM).*<br>*Extract any data related to the accountability arrangements within the improvement project (SGM/RN/FN)*	*Comment on ways in which the governance arrangements have helped the improvement process.*<br>*Comment on ways in which the governance arrangements have hindered the improvement process*
**Leadership**<br>What was the leadership style like within the steering group?<br><br>Was everyone involved in making decisions? Were difficult decisions made? Were difficult people dealt with?	*Comment on the leadership style within the steering group (SGM/RN/FN)*<br>*Comment on the way in which decisions were (or were not) reached (RN/FN), using extracted data / actual examples where possible.*<br>*Comment on your own role within the steering group*	*Comment on ways in which leadership in this site has helped the improvement process*<br>*Comment on ways in which leadership in this site has hindered the improvement process*
**Key decisions** related to the design, implementation or evaluation of the improvement project	*Insert key decisions that were made within steering group meetings and how they were made (SGM)*<br>*Extract any data related to key decisions that were NOT made within steering group meetings (SGM/RN)*<br>*Extract any data related to key decisions that were made OUTSIDE of steering group meetings (FN)*	
**Policy issues**<br>- Laws and regulations<br>- National policy<br>- Regional policy<br>- Local level policy	*Extract any data related to policy issues and the way they have affected the improvement process (SGM/FN)*	*Comment on the ways in which policy issues have helped the improvement process.*<br>*Comment on the ways in which policy issues have hindered the improvement process.*

**Organisational issues**<br>- Rules and regulations<br>- Organisation 'culture'<br>- Staff attitude	*Extract any data related to organisational issues and the way they have affected the improvement process (SGM/RN/FN)*	*Comment on the ways in which organisational issues have helped the improvement process*
		Comment on the ways in which organisational issues have hindered the improvement process
**Inter- and intra-organisation collaboration**<br>- Collaboration vs competition<br>- Division of roles & responsibilities<br>- Within organisations, between organisations, with external stakeholders<br>- Newcomers, leavers	*Extract any data related to collaboration within organisations and between organisations and the way it has affected the improvement process (SGM/RN/FN)*	*Comment on the ways in which collaboration in the improvement project has helped the improvement process*
		Comment on the ways in which collaboration in the improvement project has hindered the improvement process
**Interpersonal relations**<br>- communication<br>- partnership	*Extract any data related to interpersonal relations and the way they have affected the improvement process (SGM/RN/FN)*	*Comment on the ways in which interpersonal relations in the improvement project have helped the improvement process*
		Comment on the ways in which interpersonal relations in the improvement project have hindered the improvement process
**Availability of (non-financial) resources**<br>- information technology<br>- knowledge	*Extract any data related to the availability of (non-financial) resources and the way that has affected the improvement process (SGM/RN/FN)*	*How has the availability of (non-financial) resources helped the improvement process?*

- staff		*How has the availability of (non-financial) resources hindered the improvement process?*
**Financial issues**<br>- funding<br>- contracts<br>- within organisations or sector-wide	*Extract any data related to financial issues, including the availability of financial resources, and the way they have affected the improvement process (SGM/RN/FN)*	*How have financial issues helped the improvement process?*
		How have financial issues hindered the improvement process?
**Sustainability & transferability**<br>- Responsiveness of the steering group to feedback from monitoring/ evaluation<br>- Long-term planning<br>- Lessons that are relevant for others	*Extract any data related to sustainability and/or transferability of the improvement project, or of elements of the improvement project (SGM/RN/FN)*	*How sustainable are the improvements to integrated care?*
		How transferable are the improvements to integrated care?
