# Supplementary material for: Improving Person-Centredness in Integrated Care for Older People: Experiences from Thirteen Integrated Care Sites in Europe
Source: Int J Integr Care. 2020 Jun 26;20(2):16. doi: 10.5334/ijic.5427 (PMC7319083; doi:10.5334/ijic.5427)
Supplement: Appendix 2. — Proposition analysis framework for site-specific overarching analysis. [file ijic-20-2-5427-s2.pdf]

Appendix 2: Proposition analysis framework for site-specific overarching analysis

NAME OF SITE AND COUNTRY:

PROPOSITION	THEMATIC STATEMENTS SUPPORTING PROPOSITION	RIVAL EXPLANATIONS (THEMATIC STATEMENTS NOT SUPPORTING PROPOSITION)
1. Integrated care activities will maintain or enhance person-centredness, prevention orientation, safety, efficiency and co-ordination in care delivery	**Person-centredness**	**Person-centredness**
	**Prevention orientation**	**Prevention orientation**
	**Safety**	**Safety**
	**Efficiency**	**Efficiency**
	**Co-ordination**	**Co-ordination**
2. Explanations for		

**succeeding in improving existing integrated care initiatives will be identified**		

Notes

Building the Explanatory Model

What seems to work and with what outcomes when making improvements to integrated care?

What are the explanations for succeeding and improving integrated care initiatives?

What are the explanations for NOT succeeding and improving integrated care initiatives?

Overall Reflections

Are there any factors that are particularly strong in your analysis that could be seen as having an impact on integrated care improvements?
**What factors can you identify in your site analysis that could apply to integrated care improvements across the EU, and be transferable?**
